# Coping Mechanisms, Depression and Suicidal Risk Among Patients Suffering From Idiopathic Epilepsy

**DOI:** 10.5812/ijhrba.8621

**Published:** 2013-03-12

**Authors:** Mohsen Foroughipour, Naghmeh Mokhber, Mahmoud Reza Azarpajooh, Mina Taghavi, Morteza Modarres Gharavi, Farzad Akbarzadeh, Alireza Ebrahimi, Mehri Baghban Haghighi

**Affiliations:** 1 Department of Neurology, Mashhad University of Medical Science, Mashhad, IR Iran; 2 Psychiatry and Behavioral Sciences Research Center, Mashhad University of Medical Sciences, Mashhad, IR Iran

**Keywords:** Suicide, Epilepsy, Depression, Coping Skills

## Abstract

**Background:**

Depression disorder is the most prevalent neuropsychiatric disorder associated with epilepsy, and a correlation has been detected between depression and suicide. There is a relationship between suicidal behavior and coping mechanisms; therefore, it is important to undertake psychoanalytic psychotherapy to reduce depressive symptoms.

**Objectives:**

To evaluate the Coping Mechanisms, Depression and Suicidal Risk among Patients Suffering from Idiopathic Epilepsy.

**Materials and Methods:**

The present study is a cross-sectional pilot study in which 93 Iranian patients with idiopathic epilepsy were selected from Qaem hospital and neurological clinics. They answered three questionnaires: BDI, SSI, and a questionnaire of coping mechanisms. Patients were then interviewed and divided into two groups: patients with depression and suicidal ideation, and patients without depression and suicidal ideation. The two groups were compared in terms of coping mechanisms.

**Results:**

Among the patients who filled the questionnaires, only 74 were selected for the interview. 58.9% of the patients did not have depression or suicidal ideation and 23.3% of them had either depression or suicidal ideation. Findings of the study showed that the two groups had a significant difference in terms of repressive coping method efficiency (P = 0.022). However, there was no significant difference between the two groups in terms of problem-focused coping method (P = 0.25) and the emotion-focused coping method efficacy (P = 0.31).

**Conclusions:**

Iranian patients with idiopathic epilepsy and with either depression or suicidal ideation, make significant improvement using repressive coping method in comparison to patients with idiopathic epilepsy who did not suffer from depression or suicidal ideation. The effect of other coping mechanisms was not significantly different between the two groups.

## 1. Background

Epilepsy is a serious chronic neurological disorder which affects about one percent of the population (1, 2). There is a prevalence of approximately 700 thousand people in Iran and 60 million people around the world. A study conducted in 1990 found that the annual incidence of epilepsy in developed countries ranges from 53 to 24 per hundred thousand people (3). Epilepsy not only is a neurological disorder and a disorder of consciousness, but also it is associated with a wide range of psychological disorders. Some of them are cognitive, personality and mood disorders (4), and no doubt, depression is the most common psychiatric disorder in patients with epilepsy (5-8). Different mechanisms have been found for depression in epilepsy, and the following problems can cause depression:

1. An underlying disease of the brain which could be causing epilepsy

2. The antiepileptic drugs

3. Damages caused by repeated seizures

4. Socio - cultural constraints associated with the disorder

5. How individuals react to chronic illness

6. Patients’ obstacles in their personal lives (5).

Studies have found that depression causes disability in patients with epilepsy and reduces the patient's quality of life more than the epilepsy itself; and this is dangerous since it could lead to suicidal thoughts or suicide (9). The diagnosis of depression is an important first step for managing depression in epileptic patients. Since epilepsy as a chronic disease is difficult and sometimes impossible to cure, and also considering the fact that antidepressants could cause seizures or may interfere with antiepileptic drugs, it is important to enhance coping mechanisms in the patients. So, psychotherapy plays a crucial role in helping epileptic patients with depression (8). In this cross-sectional study, the reasons behind depression and suicidal tendencies in epileptic patients were investigated and the necessary coping mechanisms were considered.

## 2. Objectives 

The aim of this study is to evaluate the coping mechanisms, depression and suicidal risk among patients suffering from idiopathic epilepsy.

## 3. Materials and Methods

This is a cross-sectional pilot study in which 93 Iranian patients with idiopathic epilepsy with tonic colonic seizers were selected target oriented from Qaem hospital and neurological clinics.

This study has included epileptic patients, both with and without depression and suicidal thoughts, who referred to hospitals and clinics from October 2008 to September 2010. 93 eligible patients with idiopathic epilepsy were selected for study. The inclusion criteria during this study were as follows: the diagnosis of idiopathic and generalized tonic colonic epilepsy for at least one year, receiving the standard treatment (sodium valproate) with less than two seizures in month, at least 17 years of age with diagnosis of epilepsy from at least one year ago, the duration of the disease for 1-10 years, education level of primary school or higher. The exclusion criteria included: the diagnosis of psychosis, lack of any inclusion criteria, and lack of patient consent. Demographic, depressive symptoms and suicidal risks data were obtained from patients using three questionnaires: BDI, SSI, and a questionnaire of coping mechanisms. All the selected patients completed the Beck Depression Inventory (BDI) (10, 11). BDI is a questionnaire developed to measure the severity of depression including 21 multiple-choice questions to be completed by the patient. The Scale of Suicide Ideation (SSI) was measured by the experimenter. The questionnaire consists of 19 components which can be used to evaluate a patient's suicidal intentions (12), SSI was completed during the interview.

Questionnaire of coping mechanisms (10), a standard pre-formulated questionnaire, is derived from two other adaptive mechanisms questionnaires and has been standardized for the Iranian population. The questionnaire has been used in a thesis, approved by Mashhad University of Medical Sciences. This questionnaire has 60 multiple choice questions (each item scoring on a 0-4 scale). It includes three axes, each having five components:

1. Problem focused coping (problem-oriented coping); The components of problem-oriented coping are: planning, code compliance, social support as a means of overcoming isolation, resistance, and suppression of competing activities.

2. Emotion focused coping (emotion oriented coping); Components of emotion oriented coping are: positive emotions, humor, religious conformity, acceptance, use of emotion, and social support.

3. Repressive coping (Ineffective coping); Components of ineffective coping mechanisms include: intellectual fragmentation, the behavioral fragmentation, denial, substance use, and focusing on feelings.

After sampling, the samples were coded, and evaluated by SPSS software. Normally, distributed data were analyzed by Komologorov-Smirnov test. When the data is not normally distributed, Mann-Whitny test is used for two-group data and Kruskall-Wallis test is used for more than two groups.

## 4. Results

A total of 93 patients participated in the current study; 24 males (25.8%) and 69 females (74.3%). 62.4% of the patients were single, and 31.2% were married; 6.5% of them were either divorced, widowed or separated. 41.9% of the participants held a university degree. 91.3% of them had their disease under control where 8.7% of them were suffering from uncontrolled disease. Also, 5.4% of the epileptic patients had other diseases that were treated. Moreover, 6.5% of the patients had received treatment for their depression. Among patients, 50% of men and 45% of women were suffering from depression in three categories: mild, moderate and severe depression. 2.7% of the male participants and 17.6% of the females had low risk of suicidal thoughts, and 1.4% of men and 68% of women had high risk of suicidal thoughts.

**Figure 1.**
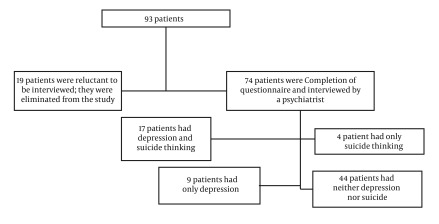
Sampling

In this study, based on the Man-Whitney test, there was no significant relationship between gender and depression (P = 0.58). Moreover, based on the above test, there was no significant relationship between gender and suicidal thoughts (P = 0.32). The Mann-Whitney test found no significant relationship between gender and disease duration (P = 0.45). 55% of the married participants and 46% of the singles had some degree of depression. 62% of the singles and 51% of the married people had no thoughts of suicide and the rest had experienced some degrees of suicidal thoughts. The Kruskal-Wallis test showed no significant relationship between marital status and BDI score (P = 0.94). Also, the test found no significant relationship between marital status and test scores (P = 0.96). According to the Kruskal-Wallis test, there was no significant relationship between marital status and duration of the disease (P = 0.35). However, the test found significant correlation between education and test scores (P = 0.15). While studying for the coping mechanisms associated with depression and suicidal thinking, 74 out of 93 patients were cooperative and conducted the interview with a psychiatrist. 12.3% of the idiopathic patients had depression only, 5.5% had suicidal thoughts or attempts, 23.3% had both depression and suicidal thoughts and 58.9% had none (Among the patients, who completed the questionnaires, only 74 patients conducted the interview. 58.9% of the patients had no depression or suicide ideation, 17.8% of the patients had either depression or suicide ideation, and 23.3% were experiencing both), (Figure 1).

Coping mechanisms were compared between the two groups with and without depression and suicidal thoughts based on the Man-Whitney test. The use of ineffective coping mechanisms between the two groups of patients is statistically significant. The average score for ineffective coping is higher in a group who had depression and suicidal thoughts (Table 1).

**Table 1.** Relation Between two Groups and Ineffective Coping Mechanisms

	Intellectual fragmentation, Mean ± SD	Behavior fragmentation, Mean ± SD	Denial, Mean ± SD	Substance use, Mean ± SD	Focusing on feeling, Mean ± SD	In-effective coping mechanisms, Mean ± SD
**Depression and suicide**						
Yes	2.1 ± 1.1	1.3 ± 0.9	0.7 ± 0.9	0.05 ± 0.2	2.1 ± 1.07	6.5 ± 2.4
No	1.4 ± 1.1	0.5 ± 0.8	1.04 ± 1.1	0.02 ± 0.1	2.09 ± 1.3	4.6 ± 3.1
**Mann-Whitney test results (P)**	0.02	0.001	0.31	0.49	0.83	0.022

Using coping strategies such as denial, substance use, coping with difficult feelings did not statistically show a significant difference between the two groups (Table 1). However, the intellectual fragmentation mechanism was significantly different between the two groups (P = 0.02); and the average score is higher in the group with depression and suicidal thoughts (2.4 ± 1.1). Also, fragmentation behavior coping mechanism was significantly different between the two groups (P = 0.001), and the average score was higher in the group who had depression and suicidal thoughts (1.3 ± 0.9). Based on the Man-Whitney test, the components of problem-oriented coping mechanisms did not statistically show a significant difference between the two groups (Table 2). Table 2 Relation Between Two Groups and Problem-Oriented Coping MechanismsAlso, according to this test, in patients with idiopathic epilepsy, problem-oriented coping mechanism is not statistically different between groups with and without depression and suicidal thoughts (P = 0.25) (Table 2). Based on the Mann-Whitney test, the components of emotion-oriented coping mechanism statistically do not have a significant difference between the two groups (Table 3). Moreover, according to this test, in patients with idiopathic epilepsy, emotion-oriented coping mechanism is not statistically different between groups with and without depression and suicidal thoughts (P = 0.31) (Table 3).

**Table 2.** Relation Between Two Groups and Problem-Oriented Coping Mechanisms

	Programming, Mean ± SD	Good Compliance, Mean ± SD	Use of Social Support, Mean ± SD	Resistance, Mean ± SD	Suppression of Competing Activities, Mean ± SD	Problem Oriented Coping, Mean ± SD
**Depression and suicide**						
Yes	1.5 ± 1.2	1.2 ± 5.9	1.9 ± 1.3	1.4 ± 1.5	0.8 ± 1.2	7.0 ± 4.3
No	2.0 ± 1.3	1.7 ± 1.3	2.2 ± 1.4	1.3 ± 1.2	1.1 ± 1.2	8.6 ± 5.1
**Mann-Whitney test results (P)**	0.30	0.25	0.46	0.93	0.40	0.25

**Table 3.** Relation Between Two Groups and Emotion- Oriented Coping Mechanisms

	Positive emotion, Mean ± SD	Humor, Mean ± SD	Religious conformity, Mean ± SD	Use of social and emotional support, Mean ± SD	Acceptance, Mean ± SD	Emotion oriented coping, Mean ± SD
**Depression and suicide**						
Yes	1.2 ± 1.1	2.8 ± 10.6	2.2 ± 1.3	1.82 ± 1.28	1.9 ± 1.4	7.7 ± 4.5
No	1.7 ± 1.1	0.2 ± 0.4	2.4 ± 1.2	1.81 ± 1.29	1.9 ± 1.2	8.3 ± 3.5
**Mann-Whitney test results (P)**	0.12	0.82	0.57	0.99	0.96	0.31

## 5. Discussion

In this study, depression and suicidal tendencies in epileptic patients were investigated and the necessary coping mechanisms were considered. 58.9% of the patients did not have depression or suicidal ideation and 23.3% of them had either depression or suicidal ideation. Findings of the study showed that the two groups had significant difference in terms of repressive coping method efficiency (P = 0.022). However, there was no significant difference between the two groups in terms of problem-focused coping method (P = 0.25), and the emotion-focused coping method efficacy (P = 0.31).

In 2002, Camfield CS et al. (13), expressed an increased risk of psycho-pathology and suicidal behavior in patients with epilepsy; Beck et al. (14), in a prospective study in 1958, described the Beck’s hopelessness scale. In a study in 2008, the authors described the importance of suicidal thoughts and coping mechanisms (15, 16). Coping mechanisms refer to the process through which a person tries to control and manage stress (17). One can think about a stressful problem or event and try to deal with their cause. This strategy is called problem-oriented coping mechanism. These individuals showed less depression during and following stressful events. One can focus on reducing the excitement associated with stressful situations. This strategy is called emotion-oriented (emotion focused) coping mechanism. Under stress, most people are likely to use a mixture of both approaches. The third strategy is called repressive coping in which the emotions are suppressed, and this approach can trigger many physical illnesses (Exacerbation) (18).

In several studies conducted by Shahid Beheshti University in Iran in 2005, Dr. Bahrinian and Dr. Karamad showed that epileptic patients have a higher prevalence of depressive disorders than the general population (17). Also, the results of a study in Isfahan University in 2006 supported the idea (19).

The results of this study indicate that the use of ineffective coping strategies between these two groups of epileptic patients is statistically significant (P = 0.022). Average score of ineffective coping mechanism use in the group with depression and suicidal thoughts was (6.5 ± 2.4) and in the other group, it was (4.6 ± 3.1). This is an indication that the ineffective coping mechanisms are used by the epileptic patients with depression and suicidal thoughts more often than the other group, and based on the obtained results, this relationship is significant and can be generalized. As mentioned earlier, components of ineffective coping mechanism include: intellectual fragmentation, behavioral fragmentation, denial, substance use, and focusing on feelings. Statistically, the use of behavioral fragmentation mechanism was significantly higher in the group with suicidal thoughts (P = 0.001). Also, the use of intellectual fragmentation mechanism was significantly different between the two groups (P = 0.02), and the average score is higher for the group with depression and suicidal thoughts (2.4 ± 1.1). However, there was no significant difference regarding other components. Regarding ineffective coping strategies, Brown et al. explained that in response to stressing factors, the autonomic nervous system of people who used ineffective coping functions more efficient than other people and this leads to great deterioration of physical health (20). Regarding substance use, there is no significant difference between the two groups (P = 0.4). Also, in terms of focus on feelings component, the average score in the group with depression and suicidal thoughts was higher.

In this study, the problem-oriented coping strategy scores between the two groups was not statistically significantly different (P = 0.25). In Billings and Moos’ study (1984), it has been determined that those who use problem-oriented coping in stressing situations are less depressed during and following the events (20), in Nezu and Perri’ study (1989), it is expressed that people who are less depressed are able to approach problem-oriented coping strategy easier (20). In the current study, although the mean score for problem-oriented coping strategy was higher in the group who had depression and suicidal thoughts, no statistically significant correlation was found (P = 0.25). Based on the research results, emotion-oriented coping strategy scores between the two groups were not statistically significantly different (P = 0.31). In a study by Hosseini *et al* (2010), coping strategies were investigated in 21 Iranian patients with epilepsy, and the results showed that these patients approached emotion-oriented coping mechanism more often than problem-oriented coping mechanism (21). 

The small sample size should be mentioned as a limitation of our study. Furthermore, to reduce the effect of interfering factors on the BDS and SSI scores, we selected our participants from the same region, i.e. the city of Mashhad. Although this makes our results more reliable, it may also raise some questions about the generalization of our ﬁndings. Therefore, conducting larger multicenter trials would be necessary to conﬁrm the results. Hence, as a conclusion, patients with idiopathic epilepsy, who had depression and suicidal thoughts, used more of the ineffective coping strategy. Also, epileptic patients who had depression and suicidal thoughts, when exposed to stressful events are more likely to suffer from intellectual and behavioral fragmentation. In sum, Iranian patients with idiopathic epilepsy and having depression or suicidal ideation used repressive coping style more often than patients with idiopathic epilepsy who did not have depression or suicidal ideation. The use of other coping mechanisms was not different between the two groups of patients.
